# Fluorescence imaging agents in cancerology

**DOI:** 10.2478/v10019-010-0031-y

**Published:** 2010-09-09

**Authors:** Aurélie Paganin-Gioanni, Elisabeth Bellard, Laurent Paquereau, Vincent Ecochard, Muriel Golzio, Justin Teissié

**Affiliations:** 1 CNRS; IPBS (Institut de Pharmacologie et de Biologie Structurale); Toulouse, France; 2 Université de Toulouse; UPS; IPBS; Toulouse, France

**Keywords:** Photonic imaging, fluorescence, cancerology, apramers, smart probes

## Abstract

**Background:**

One of the major challenges in cancer therapy is to improve early detection and prevention using novel targeted cancer diagnostics. Detection requests specific recognition. Tumor markers have to be ideally present on the surface of cancer cells. Their targeting with ligands coupled to imaging agents make them visible/detectable.

**Conclusions:**

Fluorescence imaging is a newly emerging technology which is becoming a complementary medical method for cancer diagnosis. It allows detection with a high spatio-temporal resolution of tumor markers in small animals and in clinical studies. In this review, we focus on the recent outcome of basic studies in the design of new approaches (probes and devices) used to detect tumor cells by fluorescence imaging.

## State of the art

Specific visualization of carcinogenesis or established tumor cells offers opportunities to guide surgery and monitor the response to therapy. In the clinic, radio-imaging uses contrast agents Indium-111 and Technetium-99 coupled to antibodies to target prostate^1^,^2^, colorectal^3^, ovarian^4^ or small-cell lung cancers.^5^ These radioelement-based technologies are powerful tools for the detection and therapy of cancers but they cannot be used during surgery. Fluorescence imaging is more user-friendly and provides on-line information. Therefore, fluorescence imaging agents which allow fast detection with a high spatio-temporal resolution can increase detection of the edge of the primary tumor, the presence of metastasis and therefore help tissue resection by the surgeon.

## Fluorescence imaging

### Why the NIR (near infra red) light?

In tissue fluorescence imaging, it is necessary to take into account five important parameters: reflection, absorption, refraction, background autofluorescence and distribution of photons emitted by the fluorochrome targeted to tissues. Skin is an obstacle because the emitted light is reflected by this barrier and this reflection brings a loss in the penetration of the excitation light.

**FIGURE 1:**
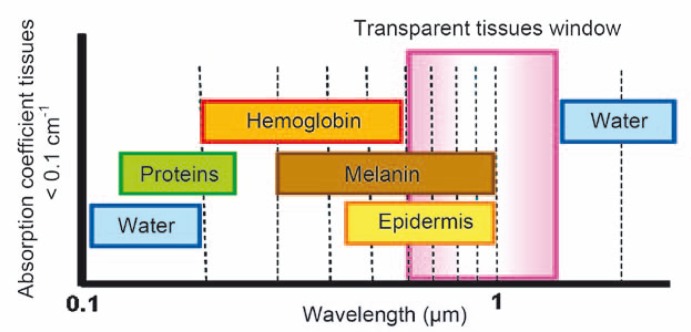
Absorption spectra of different molecules present in biological tissues. The tissue optical window (600–1200 nm) is ideally sought in fluorescence imaging of small animals. Hemoglobin and water absorb light below and above the optical window.

In tissue, different chromophores in biomolecules strongly absorb the incident (or emitted) light. This is a major limit for the near UV and visible part of the spectrum. Light absorption by hemoglobin is a problem in the visible range (from 400 to 670 nm). Indeed, the absorption coefficient (cm^−1^) decreases when the wavelength increases. Absorption due to the chromophores in biomolecules is very strong below 460 nm and remains important up to 580 nm. Thus, only a weak penetration in the tissue can be obtained. The same problem is of course present if emission is in the same wavelength range as absorption. A deeper penetration is obtained when working in the near infrared (NIR) part of the spectrum between 600 and 1000 nm (Figure 1).^6^ The upper limit in the wavelength (around 1200 nm) is due to water which is a strong light filter in IR spectroscopy.

Light scattering due to turbid media is also reduced in this high wavelength window as predicted by the Rayleigh law. Nevertheless, scattering in tissues remains high, due to refractive index mismatches between the different cellular components and fluids. This is a limit in the spatial definition.

Finally, light absorption by endogenous tissue fluorochromes can result in light emission, the so called autofluorescence of the tissue. This phenomenon is due to the oxidized forms of riboflavin, the co-enzymes flavin and NADH reduced inside cells.^7^,^8^ Other molecules like lipofuscin and ceroides or other components of the skin, such as collagen and melanin, also contribute to this effect. Autofluorescence is also a consequence of food that contains chlorophyll.^9^,^10^ Tissue autofluorescence is mainly present in the UV and visible range of the spectrum.

Compared to fluorescence imaging in the visible light range, fluorescence imaging in the NIR bandwidth offers less photon absorption by blood hemoglobin, lipid and water, and a limited light scattering, enabling photon transmission deeper into the body. Thus, substantially reduced tissue autofluorescence, enabling higher sensitivity detection of target NIR molecular imaging agents due to a low background, can be achieved.

For a greater discussion of the physics underlying efficient NIR photon delivery through tissues, fluorescence chemistry synthesis approaches and fluorescence hardware systems, the interested reader can consult several reviews.^11^,^12^

As a conclusion, an accurate quantitative and spatially resolved detection *in vivo* by an optical method faces intrinsic limitations due to the optical properties of intact biological tissues. Taking into account these optical properties of living tissues, optimized conditions by choosing the relevant biological reporter fluorophores could be obtained.

### Which fluorophore?

Two kinds of commercial organic fluorochromes emitting in the NIR wavelength domain are available: cyanine^13^ and Alexa Fluor.^14^ They can be grafted on any kind of molecules of interest such as nucleic acids, proteins or antibodies. They have several advantages such as weak toxicity, a small molecular weight, a functional group allowing their grafting and weak photo-degradation. Their limit is a weak fluorescent quantum yield.^13^ Therefore, multigrafting of these molecules on “rafts”^15^ or on dendrimers^16^ is performed to overcome this problem by increasing the local number of emitters on the target. Company brand fluorophores are now on the market (DyLight Fluor family by Dyomics in collaboration with Thermo Fisher Scientific, KODAK X-SIGHT Large Stokes Shift Dyes and nanospheres, XenoLight CF by Caliper).

Commercially available quantum dots are promising competitors of organic probes for fluorescent imaging (Qdot^®^ nanocrystals by Molecular probes, Quantum Dot Corporation Qtracker). Indeed, they have a strong fluorescent quantum yield^13^, a weak sensibility to photobleaching and a strong stability. However, they have significant toxicity *in vivo* due to their chemical core (nanotoxicology).^17^ *In vivo*, they are used with success in biphoton microscopy and some reports are cited in small animal imaging studies.^18^

## Probe design

**FIGURE 2:**
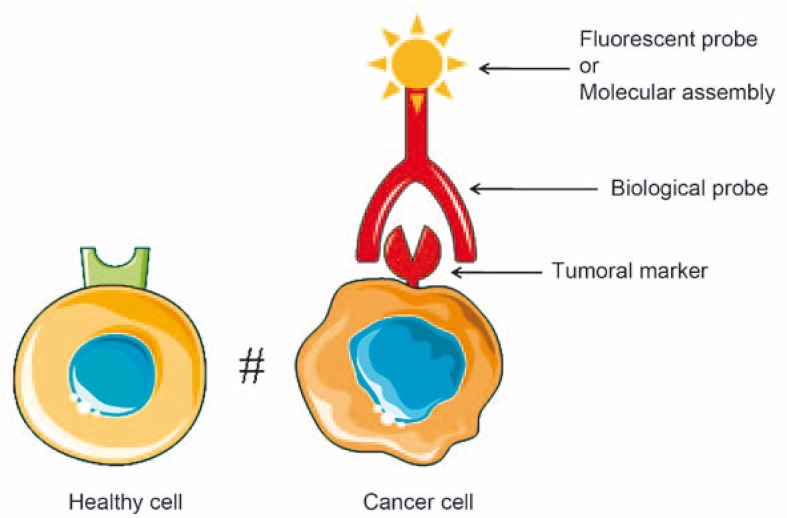
Principle of targeting tumor cells by fluorescence imaging.

Targeting tumor cells by fluorescence imaging can be achieved by coupling a fluororescent agent with biological probes (antibodies, aptamers, peptides or enzymatic ligands or metabolites) that recognize specific tumor markers only expressed or over-expressed by tumor cells. The labeling of the biological probes can be done by fluorescent markers or complex molecular assemblies (Figure 2).

### Tumor markers

Tumor cells differ from healthy cells by tumor markers which are expressed and located on their plasma membrane. These tumor markers are proteins or glycoconjugates over-expressed on the membrane surface of tumor cells such as protein receptors that interact with a panel of probes (or ligands) described in the following paragraph.

Several membrane antigens are recognized by monoclonal antibodies and used for imaging of tumors: prostate specific membrane antigen (PSMA) 16,19,20, the carcino embryo antigen (CEA)^21^, the VEGF receptor (*Vascular Endothelial Growth Factor*) or the Human Epidermal Growth Factor Receptor-2 (HER-2).^22^,^23^ These antigens are membrane proteins over-expressed by tumor cells and involved in life processes such as exogenous or endogenous transduction of signals or the cell cycle. In addition, they can be used in imaging to detect various tumors.

PSMA is a membrane and cytoplasmic glutamate carboxypeptidase which is involved in the cell cycle and in carcinogenesis associated with prostate cancer. CEA is involved in cell adhesion and is found in various cancers such as colorectal, gastric, pancreatic, lung and breast cancers. The VEGF receptor is over-expressed in most tumor and endothelial cells involved in angiogenesis. HER-2 is a tyrosine kinase membrane receptor involved in signal transduction pathways inducing growth and cellular differentiation. It is over-expressed in breast and ovarian cancers and other carcinomas.

There are other proteins over-expressed on the surface of several types of tumor cells such as metalloproteinase-2 (MMP-2) 24, integrins αVβ3 25,26 and lectins.^27^ These molecules are less specific for tumor cells than the antigens described above because they are also expressed by healthy cells but in much smaller quantities. Their natural ligands are used as probes.

### Biological probes

#### Antibodies

Antigenic tumor markers used in molecular imaging are generally derived from anatomopathological tissues studies. A large library of antibodies specific for tumor cells has been gathered. They have been adapted for human administration (humanized and recombinant antibodies). Monoclonal antibodies are widely used in fluorescence imaging due to their strong affinity for their target. On the other hand, one should keep in mind their disadvantage of triggering immune reactions. It is difficult to find a good compromise between modifications (humanization, chimerization) of antibodies to make them more biocompatible and their loss of affinity for their target. Several monoclonal antibodies are available for *in vivo* fluorescence imaging applications: the anti-PSMA antibody that targets prostate tumor cells^20^,^28^, the anti-CEA antibody that targets tumor cells of prostate, pancreas and colorectal cancer^29^,^21^, the anti-VEGF receptor antibody that targets tumor cells and those associated with the angiogenic process^30^ or the anti-HER-2 targeting tumor cells in breast, ovary, and other carcinomas (Table 1).^23^,^31^

#### Peptides and proteins

Peptides or proteins can also be used to target tumor cells but they are still at an experimental stage. This approach consists of using the binding properties of the peptide (or protein) with glycoconjugates or membrane proteins over-expressed in tumor cells (Table 1). For example, Chlorotoxin is used to detect various tumor cells (glioma, medulloblastoma, prostate cancer, bowel cancer and sarcomas).^24^ This peptide, derived from scorpion venom, is composed of 36 amino acids with 4 disulfide bonds and interacts with MMP-2. Due to its anti-cancer properties, it can be used to target tumor cells. *In vivo* detection of cells over-expressing MMP-2 was obtained by non-invasive fluorescence imaging.^24^ Cyanine 5.5 was coupled to primary amines of Chlorotoxin (3 amino functions). Another example is the RGD peptide, which is a cyclo-peptide that mimics angiotensin. It is used to detect tumor cells because it specifically interacts with α_V_β_3_ integrins over-expressed on the surface of many different tumor cells.^25^,^26^

#### Metabolites

Another approach is to use metabolic properties of tumor cells that differ from normal cells. Indeed, they absorb more nutrients because they over-express proteins involved in cell growth. Thus, administration of metabolites is used to target receptors over-expressed in tumor cells (Table 1). For example, albumin that interacts with the β-D-galactose receptor 27 or folic acid (vitamin B9) that interacts with the folate receptor (or folate-binding protein (FBP) 32,33, are both effective for locating various tumor cells (ovary, kidney, uterus, brain, colon, lung adenocarcinoma). This approach is less specific for tumor cells than approaches targeting tumor antigenic markers but it is widely used in imaging modalities such as MRI and PET for the specific detection of tumor cells and also for drug-targeted delivery to tumors.

#### Aptamers

Aptamers can be used for targeting live cells. Aptamers are highly structured oligonucleotides selected by Systematic Evolution of Ligands by Exponential Enrichment (SELEX) to bind tightly (nanomalor range) and specifically to a target molecule. Recently, specific aptamers have been selected against tumor markers like PSMA or MUC1 (Table 1).^19^,^34^

Nucleotidic aptamers present all characteristics, which make them suitable as imaging probes: they are smaller (10–15 kDa) than antibodies (150 kDa), hence they exhibit higher tissue penetration and faster blood clearance. In addition, compared with antibodies, aptamers present a low immunogenicity, are not toxic and they can be chemically modified.^35^

The first aptamer used in imaging was designed against human neutrophil elastase.^36^ This work demonstrated for the first time the potential feasibility of using an aptamer labeled with technetium-99m (^99m^Tc) as reagents for diagnostic imaging. The aptamer had a signal-to-noise ratio higher and more rapid than the antibody.

More recently, an aptamer labeled with ^99m^Tc directed against human tenascin-C was also used for *in vivo* imaging.^37^ These authors showed a rapid uptake of aptamers by tumor and a rapid clearance from blood and other non-target tissues, which enabled clear tumor imaging.

Another report used ^99m^Tc -labeled-aptamer directed against MUC1 and was tested in MCF-7 tumor-bearing mice.^38^ Their first results showed the necessity to optimize the radiolabeled aptamer in terms of pharmacokinetics prior to use in imaging.

Actually, fluorescently-labeled aptamers that bound the tumor cell surface were either used for *in vitro* imaging on culture cells that expressed, for example, PSMA^39^ and MUC1^34^, or by injections of a fluorescent aptamer against tenascin-C into tumor-bearing mice followed by fluorescence microscopy on tissues sections.^37^ However, they are still not often used in fluorescence imaging of small animals.

#### Smart probes

“Smart probes” or “smart sensors” are probes activated by an intracellular proteolytic reaction of targeted tumor cells that become fluorescent. These probes give an excellent signal-to-noise ratio because they are activated only when internalized in target cells. Basically, they are activated by proteases or intracellular reductases (metalloproteinases MMP-2, cathepsins B and D, cysteine proteases, thioreductases) over-expressed in tumor cells which cut Lys-Lys or disulfide bonds of the complex and release the fluorophore.^35^,^40^

ProSense probes developed by Weissleder (VisEn Medical, Inc., Woburn, MA) are polylysines labeled by non-fluorescent cyanines. When the probe is internalized into cells by endocytosis, the peptide link (between lysines) separating the cyanines is broken by the action of intracellular proteases such as cathepsins (B or D) or metalloproteinases (MMP-2) and fluorophores are released into the cells which become fluorescent.^24^,^41^

Razkin *et al.* have shown that the molecule RAFT-RGD-Cy5-SS-Q penetrates effectively and specifically in tumor cells and is activated once inside. The complex consists of 4 RGD peptides specifically targeting the αVβ3 receptors over-expressed on the surface of cancer cells, and of a quencher (Q) connected to a cyanine 5 via a disulfide bond. This bond is reduced by thioredoxin in the cytoplasm and endosomes after internalization into cells. Once internalized, the quencher is spatially separated from the cyanine and the complex becomes fluorescent. The phenomenon of quenching can be achieved by combining two identical fluorophores but the rate of cleavage of the disulfide bond is weaker and the contrast obtained *in vivo* is much smaller.^40^

Engelman *et al.* demonstrated that the pH low insertion peptide (pHLIP) is able to insert into the lipid bilayer of the plasma membrane by forming an α helix when the acidity increases in the extracellular matrix.^42^ Indeed, the extracellular matrix surrounding tumors and areas of inflammation or infection are relatively acidic environments compared to healthy tissues. The insertion of the peptide in the cell membrane occurs at a pH below 6.5. The C terminal end of the complex is trans-located into the cytoplasm. Two applications are then possible — the targeted delivery of drugs in tumor cells and the fluorescence imaging of these cells. Engelman *et al.* first grafted a disulfide bond to the C terminal end of the peptide, linking it to a fluorescent molecule or a drug that can be released into the cells by cleavage of the disulfide bond by thioredoxin. They also showed that this peptide is effective *in vivo* for detection of tumor cells by noninvasive fluorescence imaging. It is shown that this peptide localizes specifically in tumor cells within 20 hours.^43^

#### Molecular assemblies

Functionalization of fluorescent agents by coupling with enzymatic ligands^44^, antibodies^45^ or peptides^46^, enable their targeting to tumor cells. Classically, tumor probes are bound to an organic fluorophore^45^,^21^ or quantum dot^28^,^31^ to visualize tumor cells by fluorescence imaging. The commercial fluorophores have reactive groups such as amine, carboxylic acid or thiol of the amino acid of the protein probe. However, the number of reactive groups per probe is low. According to protein size and the number of reactive groups, 4 to 10 fluorophores can be grafted per protein. In order to increase the fluorescence signal of tumor probes and/or increase their specificity for target cells, molecules called “platforms” were used as a covalent support to several fluorophores and/or several probes (Table 1).

The quantum dots can be used as “platforms” because they allow several connections with biological probes. Cai *et al.* have shown this with the RGD peptide by grafting multiple RGD peptides onto a quantum dot.^26^ This greatly increases the specificity of quantum dots for tumor cells.

The work of Coll *et al.* on the regioselectively addressable functionalized template RGD peptide (RAFT RGD) showed the specific labeling of tumor cells over-expressing integrin αVβ3 receptor. This molecule is a deca-peptide accepting 4 cyclo-RGD peptides and one fluorochrome of the cyanine 5 type. They showed that it was necessary to have at least 4 RGD peptides per platform to specifically detect tumor cells *in vivo.*^44^

Dendrimers are now experiencing their first major applications as diagnostic agents when grafted with contrast agents^47^,^48^ or fluorochromes^23^,^20^,^49^ and targeting agents. PAMAM dendrimers are used in imaging because they are water-soluble, biocompatible and biodegradable.^50^,^51^ They allow an increase in the sensitivity of detection because several imaging agents are bound per dendrimer. Furthermore, by increasing the number of biological probes by complex, it is possible in some cases to increase the specificity of the detection signal. The work of Hill and that of Thomas show the detection efficiency in fluorescence imaging of tumor cells *in vivo* by a complex composed of a PAMAM dendrimer with multiple RGD peptides^52^,^53^ and several fluorochromes.^52^,^49^ This approach can increase both the fluorescence signal of tumor probes and their specificity for tumor cells. Several studies using dendrimers as imaging agents are reported in Table 1.

Dendrimers are real molecular platforms that may also be grafted to drugs. These systems allow us to specifically target cells and provide local delivery of drugs in patients.^54^

## Conclusion

In small animals, optical imaging is a low-cost technology by which tumor cells are detected over several weeks depending on the mouse strain. Whole-body imaging gives access to relative quantitative detection with a “crude” topological definition over a long period. The technology is rather simple and is now available on the market (Berthold, www.bertholdtech.com; Hamamatsu, www.hama-comp.com; Caliper Xenogen, www.caliperls.com; Fuji, www.fuji-sciences.com; Carestream, www.carestreamhealth.com; Cambridge Research Instrumentation, www.cri-inc.com; Biospace, www.biospacelab.com). This was recently reviewed as a technological feature in “Nature”. Detection is associated with a light signal. The major limit is sensitivity and topological definition which remains associated with the turbidity of tissues. It could be improved by selecting the probes, light source and detector suitable for red fluorescence detection to avoid tissue absorption. More accurate data is obtained by other methods (intravital microscopy) but over a more limited period of time due to the associated surgery (Cellvizio, www.visualsonics.com; macrofluo, www.leica-microsystems.com; macroscope, www.nikoninstruments.eu). Real-time imaging of tumors by an IV injected probe sensitive to angiogenesis (AngioStamp^®^, Angiosense), can be obtained by a user-friendly intra-operative imager (Fluobeam^®^) that will drastically improve cancer surgery. Preclinical devices are available. The new (“smart”) fluorescent probes associated with fluorescence endoscopy should help surgeons with tumor resection in the near future (Fluoptics, www.fluoptics.com; Visen, www.visenmedical.com). A preclinical study just showed a better survival over a 6-month period when tumors in the animal were resected by using a Cy5-labeled cell-penetrating peptide conjugated to a dendrimer to guide surgery.^56^

Therefore, following and quantifying tumor progression *in vivo* by optical imaging is a fantastic tool to monitor the expression of therapeutic genes in target tissues, in disease models and/or to assess the effectiveness of cancer therapies (surgery, radiotherapy, gene therapy). Added to the routinely used imaging techniques^57^,^58^, it can be used for diagnostic evaluation and surgical management.

## Figures and Tables

**TABLE 1:** Examples of tumors markers and probes used in fluorescence imaging

**Tumor markers**	**Probes**	**Fluorescence and platforms**	**Cancers**	**References**
PSMA	Antibody	PAMAM + (x6) rhodamine or (x6) FITC	Prostate	20, 28
PSMA	Aptamer	Rhodamine, QDots	LNCap	19, 39
CEA	Antibody	AlexaFluor 488; Cyanine (DY-676)	Colorectal, gastric, pancreatic, lung, breast	21, 29
HER-2	Antibody	PAMAM + (x5) AlexaFluor 488	Breast, ovarian carcinoma	23, 31
VEGF Receptor	Antibody	NIR-800 Licor	Brain	30
Integrin α_V_β_3_	RGD Peptide (c(RGDyK); RGD-4C (doubly cyclised RGD); c(RGDfK))	Q-Dot 705; PAMAM +(x3) Alexa Fluor 488; PAMAM +(x4) FITC; RAFT +(x2) Cy5	U87MG, brain, HUVEC, HEK293	26, 49, 52
β-D-galactose receptor (lectin)	BSA / GSA	Rhodamine G	Ovarian and adenocarcinoma	27
MMP-2	Chlorotoxin	Cyanine 5.5	Glioma, neuroectoderma	24
Folate receptor	Folate	Q-Dots	Brain	32, 33
Mucine MUC1	Aptamer	Rhodamine	MCF7	34
